# The Therapeutic Effect of the Chinese Herbal Medicine, Rehmanniae Radix Preparata, in Attention Deficit Hyperactivity Disorder* via* Reversal of Structural Abnormalities in the Cortex

**DOI:** 10.1155/2018/3052058

**Published:** 2018-10-14

**Authors:** Haixia Yuan, Meng Yang, Xinmin Han, Xinqiang Ni

**Affiliations:** ^1^Nanjing University of Chinese Medicine, First Clinical Medical College, Institute of Pediatrics of traditional Chinese Medicine, Qixia District, Nanjing, 210029, Jiangsu Province, China; ^2^Nanjing University of Chinese Medicine, Institute of Chinese medicine literature, Qixia District, Nanjing, 210029, Jiangsu Province, China; ^3^Shenzhen traditional Chinese Medicine Hospital, Pediatrics of traditional Chinese Medicine, Shenzhen, 518038, Guangdong Province, China; ^4^Institute of Geriatrics, Shenzhen, 518035, Guangdong Province, China

## Abstract

Rehmanniae radix preparata is extracted from wine-steaming the Rehmannia root, a scrophulariaceae plant. It has been used for thousands of years with effects of nourishing kidney-yin, benefiting essence and filling marrow based on traditional Chinese medicine (TCM) theory. Rehmanniae radix preparata has antioxidant, antisenescence, anti-inflammatory, and neuroprotective properties. It is the most popular Traditional Chinese medicinal compound (TCMC) used in attention deficit hyperactivity disorder (ADHD) therapy. However, few studies have been conducted exploring the effects and potential mechanisms of Rehmanniae radix preparata alone on ADHD. Recent studies have shown that Rehmanniae radix preparata inhibits spontaneous activity in mice, improves learning and memory in rats following thalamic arcuate nucleus injury, and exhibits antidepressant effects. Catalpol, an active component of Rehmanniae radix preparata, elevates brain-derived neurotrophic factor (BDNF), and attenuates neuronal apoptosis and energy metabolism failure. ADHD is characterized by hyperactivity-impulsivity and impairments in learning and memory. Its pathomechanism is closely related to structural abnormalities in the cortex that is mediated by dysfunction in neuronal development, apoptosis, and energy metabolism. We hypothesize that Rehmanniae radix preparata may be effective at treating ADHD by alleviating neurodevelopmental abnormalities, neuronal apoptosis, and energy metabolism failure.

## 1. Introduction

Attention deficit hyperactivity disorder (ADHD) is the most common cognitive and behavioral disorder diagnosed during childhood, and often continues into adolescence and adulthood. The worldwide prevalence of ADHD is 3.4% [1]. It is characterized by deficient attention and problem solving, along with hyperactivity and impulsiveness, coexisting with anxiety, depression, and antisocial behavior. ADHD is a complex and heterogeneous disorder and its etiology is not fully understood. Multidisciplinary studies have provided evidence that genetic factors have an important role in its etiology [2]. Additionally, environmental risk factors, such as biological adversity (e.g., exposure to toxins, pregnancy, delivery complications) and psychosocial adversity (e.g., maltreatment and emotional trauma) are etiologically correlated with ADHD [3]. Studies have linked ADHD to higher criminal activity and suicide [4]. Methylphenidate (MPH) is a first-line therapeutic psychostimulant drug that significantly improves inattention, hyperactivity, and impulsivity [5]; however, some evidence suggests that it can induce obsessive–compulsive behavior [6], depression, and anxiety [7]. These side effects have restricted clinical application of MPH; therefore, safe and effective alternative therapeutics are required.

ADHD is characterized by structural abnormalities in brain, especially the cortex [8–10]. Abnormal cortical structure may involve neuronal development, apoptosis, energy metabolism disorder and mitochondrial dysfunction [11]. It is noteworthy that these findings about pathogenesis are in line with the traditional Chinese Medicine (TCM) theory of ADHD, which is characterized by systematology and holism. This improves the scientific hypothesis that TCM can have therapeutic benefits in ADHD, and suggests that TCM is a promising alternative treatment for ADHD due to its multitarget therapeutics with higher efficacy and lower toxicity.

In TCM theory, the brain is defined as “the house of spirit” and “sea of marrow.” The brain marrow is the material basis for the generation of spirit. The brain is formed from marrow that originates from essence stored in the kidney. Thus, the kidney stores the mind, which is related to memory, consciousness, and thinking. TCM therapy supplements the kidney essence, replenishes marrow, and shows positive efficacy and safety in treating neurological and mental diseases, including ADHD [12]. TCM therapy may ameliorate learning disorders, memory, and cognitive dysfunction by improving cellular energy metabolism and utilization, activating endogenous neurotrophic factors, and decreasing neurotoxin production, thus reducing cell death and increasing neuronal survival and regeneration [13]. Data mining shows that Rehmanniae radix preparata is the most frequent Chinese herbal medicine used to treat ADHD [14].* Bencao Tujing* (*Commentaries on the Illustrations*) states that Rehmanniae radix preparata, extracted by wine-steaming the root of Rehmannia, can benefit kidney essence, fill marrow, and enrich the blood.

**Figure 1:**
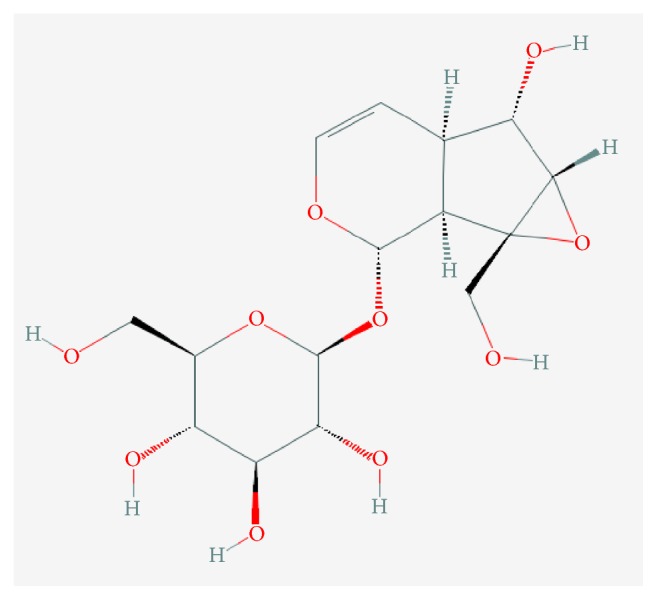
Chemical structure of Catalpol.

Rehmanniae radix preparata possesses several pharmacological properties, such as central inhibition [15], antioxidative [16], antiaging [17], and those that enhance immunity [18]. The level of catalpol (Figure 1), an iridoid glycoside, in the extract is an indicator of the quality of Rehmannia according to* Pharmacopoeia of China (2010 version).* Its molecular formula and weight are C_15_H_22_O_10_, and 362.33, respectively. Extensive research [19] shown that catalpol exerts effects in patients with Parkinson's disease (PD), Alzheimer's disease (AD), cerebral ischemia (CI), and neural senescence* via* neuroprotection, alleviating energy metabolism failure, and preventing neuronal apoptosis. These properties correlate with the pathogenesis of ADHD. Taken together, we deduced that Rehmanniae radix preparata is a potential therapeutic herb resource for ADHD treatment. Our hypothesis is discussed in this review.

## 2. Structural Abnormalities in the Cortex in ADHD

ADHD has been linked to dysfunction in several systems, including the dopaminergic [20], adrenergic [21], serotoninergic [22] and cholinergic [23] pathways. ADHD is a neurodevelopmental disorder and alterations in these neurotransmitter systems may not fully explain the complexity of ADHD. Neuroimaging has suggested that structural abnormalities in the cortex are related to the symptoms of ADHD [11, 24]. One study has reported that asymmetric structure and function in the prefrontal cortex and decreased regional cerebral blood flow to the right prefrontal cortex in children with ADHD [25]. Children with ADHD show globalized thinning of the cortex, prominently in the medial and superior prefrontal and precentral regions [26, 27]. The median age of peak thickness for 50% of regions in children with ADHD was 10.5 years, which was significantly later than typically developing children (median age, 7.5 years). This suggests that there is a marked maturational lag in cortical development in ADHD [8].

Other key morphologic features associated with ADHD include significant bilateral decreases in surface area and cortical folding, which results in a smaller cortical volume in children with ADHD [28]. Tomohiro Nakao and colleagues found that people with ADHD had a significant reduction in grey matter volume but delayed development progression could be improved with advancing age and stimulant medication [29]. One study has shown a significant reduction in thalamic volume and the dorsolateral prefrontal cortical area in both hemispheres of children with ADHD [30]. Furthermore, their results revealed a direct association between thalamic and frontal anatomy in ADHD, and suggested that the pathophysiological alterations in cortex maturation might be linked to the development of the thalamus. Other regions also display structural abnormalities in ADHD, such as reduced striatum size, which is related to hyperactivity and working memory performance [31], and a reduction in cerebellar volume, which is associated with cognitive and affective processes [32]. Multifaceted morphological alterations in brain regions are consistent with the clinical heterogeneity of ADHD. Studies have revealed that a neuronal development disorder is essential to mediate the structural abnormalities in cortical [33]. In addition, multiple molecular processes play a role in dysfunctional neuronal development in ADHD [34].

### 2.1. Disrupted Transcription Factors and Regulatory Proteins

Transcription factors (TFs) and regulatory proteins (RPs) have timing-critical roles in cortex formation, including proliferation, migration, guidance targeting, and connectivity of neurons. It is widely accepted that classical neurotransmitters, such as dopamine and serotonin, play an early role in controlling neuron number within the prefrontal cortex (PFC). During critical phases of brain development, dysregulation of serotonin and dopamine can have significant consequences for the development of disorders of the PFC, and mediate cognitive impairment [34–37].

Brain-derived neurotrophic factor (BDNF) is associated with neurogenesis and neuroplasticity in the brain. It is essential for the regulation of neuronal growth, differentiation, and survival [38]. Conditional BDNF knockout mice exhibit hyperactivity [39]. MPH enhances murine neuronal maturation and balances the retardation of neuronal development* via* the up-regulation of BDNF [40, 41].

Tbr2 is required for the specification and proliferation of intermediate progenitor cells in the cerebral cortex during brain development. Reduced Tbr2 expression results in a reduced cortical surface and thickness [42, 43]. In addition, several fibroblast growth factors (FGFs) and receptors (FGFRs) are involved in the regulation of neuronal growth and patterning, axon outgrowth, and myelinogenesis [44]. FGF2 knockout mice exhibit hyperactivity [44]; dysfunctional FGFR1 signaling is associated with spontaneous hyperactivity [45]; and FGFR2 deficient mice display hyperactive behavior [46].

Cyclin-dependent kinase 5 (Cdk5) is a neuronal serine/threonine protein kinase critical to proper neuronal migration, corticogenesis, and the regulation of postsynaptic dopamine signal integration. Mice deficient in Cdk5 display severe defects in cerebral cortex formation [47, 48]. Mice lacking p35, a neuronal specific activator of Cdk5, display severe cortical lamination defects [49, 50], and p35^−/−^ mice exhibit significant hyperactivity [51].

N-methyl-D-aspartate receptors (NMDARs) are involved in the maturation of cortical neurons and in early synapse formation [52]. NMDAR antagonists induce attentional deficits and impulsivity [53]. Neuregulin 3 (Nrg3) is critical for the development of the embryonic cerebral cortex as it regulates cortical cell migration and patterning. Nrg3 knockout mice display induced hyperactivity and deficient fear conditioning [54].

In summary, neurodevelopmental disorder-associated factors are involved in cortical development and maturation and sculpt the identity of cortex. Much evidence has shown that disturbed TFs and RPs are significant factors in the complex pathogenesis of ADHD.

### 2.2. Mitochondrial and Energy Metabolism Dysfunction and Neuronal Apoptosis

Mitochondria play a crucial role in (adenosine triphosphate) ATP production* via* oxidative phosphorylation, which is carried out by the respiratory chain complexes I, II, III, and V [55]. Additionally, mitochondria are associated with apoptosis because they contain and release proteins involved in the apoptotic cascade, such as cytochrome c and apoptosis inducing factor [56]. Mitochondrial dysfunction contributes to neurodegenerative diseases and psychiatric disorders* via* neuronal apoptosis and impaired energy metabolic processes in the brain [57]. In the spontaneously hypertensive rat (SHR) model of ADHD, Doroshchuk and colleagues observed deficient mitochondria function in the brain, decreasing the ATP synthesis rate by 30%, when compared with Wistar-Kyoto (WKY) rats, which is considered as the main cause of cell energy deficiency [58]. The mitochondrial energy metabolism generates reactive oxygen species (ROS) that mediate important physiological activities, such as cell growth and differentiation. However, excessive generation of ROS disturbs the intracellular antioxidant defense systems, leading to an imbalance in redox homeostasis, which leads to neuronal cell death associated with ADHD [59]. An increased oxidative and nitro-oxidative stress and decreased antioxidant capacity of the brain are key factors involved in the etiology of neuropsychiatric diseases. A recent advance has been the discovery that SHR presents an oxidative profile that is characterized by an increase in ROS production without an effective antioxidant counterbalance in the cortex, striatum, and hippocampus [60]. Moreover, both acute and chronic use of MPH in SHR induces oxidative damage in multiple brain regions* via* increased lipid peroxidation and protein carbonylation and reduced antioxidant enzymatic systems, such as superoxide dismutase (SOD) and catalase (CAT) [61].

Some neuropsychiatric disorders, including ADHD, can be viewed as cortical oxygen-dependent energy-deficit syndromes [62]. Sufficient cerebral blood flow (CBF) is essential for energy production; however, children with ADHD exhibit reduced CBF in the prefrontal cortex [63]. Attention is dependent upon a continuous energy supply for efficient and consistent function; therefore, reduced blood flow will impact attention processes. The astrocyte-neuron lactate shuttle (ANLS) is the primary source of energy to fuel sustained neuronal activity [64]. Patients with ADHD exhibit astrocytic dysfunction, such as in the formation and supply of lactate. This results in an inadequate supply of ATP to meet the high-energy demands of rapidly firing neurons and impaired frontostriatal communication. Furthermore, an insufficient lactate supply in oligodendrocytes impairs fatty acid synthesis and the myelination of axons during development [65, 66].

Evidence suggests that there is a maturational delay in the development of frontostriatal neural networks in the SHR that is similar to the developmental delay seen in children with ADHD [67], which is the result of diminished energy supply [68]. This study showed that the SHR has reduced protein levels involved in energy metabolism, neurotransmission, cytoskeletal structure, and myelination when compared with WKY rats, suggesting that the SHR has a diminished capacity to generate ATP. Interestingly, MPH treatment can impact the energetic metabolism in the brain in several ways. Chronic exposure to MPH activates the mitochondrial respiratory chain by increasing mitochondrial enzymes, such as succinate dehydrogenase, complex II, IV, and creatine kinase, which are involved in energy homeostasis [69, 70]. Nevertheless, other studies have demonstrated that MPH treatment leads to abnormalities in Krebs cycle enzymes and mitochondrial dysfunction, reduced citrate synthase (CS) and isocitrate dehydrogenase (ID), and inhibition of complexes I, II, II–III, and IV [61, 71]. However, a different study suggests that MPH decreases ATP levels without altering respiratory chain complex activities in juvenile rat hippocampus [72].

Few studies have been carried out to validate the connection between apoptosis signaling pathways and ADHD. Strikingly, as a first-line therapeutic drug of ADHD, MPH activates the initial cascade of apoptosis, increasing Bax and reducing Bcl-2; however, it also inhibits apoptosis by reducing caspase-3 and cytochrome c [73]. One study has shown that caspase-3 deficiency results in disrupted synaptic homeostasis. Furthermore, caspase 3^−/−^ mice exhibit reduced attention control, increased impulsive behavior, and hyperactivity [74].

## 3. Kidney Essence and Brain Marrow Deficiency and ADHD

### 3.1. Etiology and Pathogenesis of TCM Theory

According to TCM theory, the yin and yang are summarized as the two opposite aspects of interrelated items or phenomena in nature. The yin masters calmness and the yang masters movement. Yin-yang theory holds that the yin and yang are opposing and constraining; interdependent and mutually promoting each other. Physiologically, normal life activities of the human body result from the coordination between the yin and yang in a unity of opposites. Additionally, the yin and yang wane and wax in relation to each other; the waning of yin will lead to waxing of yang and* vice versa*. TCM theorizes that if this relationship changes beyond normal limits, the dynamic balance of yin and yang will not be maintained, and result in an excess or deficiency of yin or yang and the occurrence of diseases.

The theory of TCM argues that the kidney stores the essence that governs the mind. This kidney essence comes from parents and replenished by food-essence that is transformed by the spleen and stomach. Physiologically, the kidney contains insufficient essence in children, leading to immature function. Pathologically, congenital factors, such as premature labor, dystocia, injuries during birth, and low birth weight, are primary risk factors for a deficiency in kidney essence and can induce a reduction in blood flow, which may result in inadequate CBF related to ADHD. The literature examining the association of ADHD with pregnancy and delivery supports the idea that these adverse events can predispose children to ADHD [75].

The essence pertains to yin; therefore, a deficiency in kidney essence may lead to a relative and pathogenic exuberance of yang. This may affect mental activities and cause hyperactive and impulsive behavior. TCM theory states that the kidney also manufactures marrow, which originates from essence, gathers in and nourishes the brain ultimately. The brain is seen as the “the sea of marrow” and “the house of spirit” in TCM theory. It is associated with memory, consciousness, and thinking; therefore, a deficiency in kidney essence can lead to a reduction in debilitation brain marrow, resulting in attention deficits. Importantly, TCM theory argues the close connection between kidney essence, marrow, and the brain. Inadequate storage of kidney essence is the key pathogenesis in ADHD. This theory is supported by a cross-sectional retrospective study from Li and colleagues [76] who explored TCM syndrome characteristics in 170 children with ADHD. They found that the most common syndrome (*n *= 72) was a “deficiency in kidney essence and marrow in the brain”. Ma and colleagues first proposed the hypothesis that there might be a maturational lag in the development of brain marrow, which was implicated in ADHD. They proposed that the deficiency in kidney essence, maturational retardation of marrow, and imbalance of yin and yang were the pathomechanisms of ADHD [77]. This hypothesis is congruent with the theory that the maturational delay of cortical regions is involved in the pathogenesis of ADHD.

### 3.2. Targeting the Kidney Essence and Brain Marrow

Based on the syndrome differentiation, the deficiency in kidney essence and brain marrow suggests that ADHD therapy should supplement the kidney and replenish the essence. Chinese herbal medicine (CHM) formulae including prescriptions of several herbs, animal drugs, or minerals, to supplement the kidney and replenish the essence have been applied to clinical treatments of ADHD, for example, Yizhi Ningshen Granule, consisting of Rehmanniae radix preparata, Polygalae radix, and Rhizoma Acori Tatarinowii. In a randomized trial of 8 weeks treatment, children with ADHD (n = 55) treated with Yizhi Ningshen Granule had a higher effective percentage (90.91%) when compared with the MPH-treated group 10–20 mg·d^−1^; n = 51, 86.27%), with few side effects [12]. Similarly, Pediatric Huanglong Granule [78], Xiaoer Zhili Syrup [79], and Yizhining decoction [80] can supplement the kidney and replenish essence with positive efficacy and safety in children with ADHD. These treatments can significantly alleviate syndromes such as hyperactivity, restlessness, rhembasmus, loquacity, and quick-tempered behavior. It is noteworthy that most of these formulae share the same primary ingredient: Rehmanniae radix preparata.

### 3.3. The Modern Biological Basis of Supplementing the Kidney and Replenishing Essence

Li has interpreted the modern biological basis of “kidney manufacturing marrow and the brain as a sea of marrow,” proposing that the nature of “brain marrow” is neurons and neurotrophic factors in the brain. A “deficiency in brain marrow” is caused by atrophy, loss of neurons, and decreased neurotrophic factors in the brain, resulting in cognitive impairment and neurodegenerative diseases, such as dementia. The modern biological basis of “supplementing the kidney to replenish marrow” includes improving the neuronal energy metabolism and utilization, enhancing endogenous neurotrophic effects, and decreasing the production of neurotoxins, therefore, reducing cell death and increasing the survival and regeneration of neurons [81]. CHMs are characterized by multitargeted treatment to “supplement the kidney and replenish marrow.” These treatments could be useful against the complex pathogenesis of AD by exerting neuroprotective, neurotrophic, and regenerative effects, and protective effects on mitochondria and synapses [82–85].

## 4. Rehmanniae Radix Preparata and Catalpol

There are many effective CHM formulae used by TCM physicians to treat the different syndromes of ADHD in children [86]. We performed a recent literature by data mining the public periodical literatures from 1999–2014 [14]. In this study, we found 88 different CHM formulae, containing a total of 123 herbs, were used. The most frequent 12 herbs (frequency ≥20) were Rhizoma Acori Tatarinowii, Polygalae radix, Rehmanniae radix preparata, Os Draconis, Glycyrrhizae radix et rhizome, Poria cocos, Concha ostreae, Testudinis carapacis et plastri, Paeoniae Radix Alba, Schisandra chinensis, Corni fructus, and Dioscoreae Rhizoma. Intriguingly, five of these herbs (Rehmanniae radix preparata, Testudinis carapacis et plastri, Schisandra chinensis, Corni fructus, and Dioscoreae Rhizoma) are beneficial for kidney essence. Association rules analysis revealed that Rehmanniae radix preparata was the most frequently used herb (159/268), and had a strong correlation with other herbs. Therefore, we argue that Rehmanniae radix preparata may be a key herb in the treatment of ADHD.

Rehmanniae radix preparata can improve learning and memory function in rats with injured thalamic arcuate nuclei, induced by monosodium glutamate (MSG), by increasing the expression of hippocampal c-fos and nerve growth factor (NGF) [16]. Rehmanniae radix preparata has obvious antioxidant properties; it increases the activity of SOD and decreases the amount of malondialdehyde (MDA) in the brains of D-galactose-induced senile rats, leading to an amelioration of learning and memory function [17]. Both Rehmanniae radix preparata and its polysaccharide inhibit spontaneous activity in mice [15]. Catalpol is an active constituent of Rehmanniae radix preparata, and an indicator of the quality of Rehmannia. Catalpol can alleviate memory deficits by elevating BDNF of an animal model of Alzheimer's disease, induced by beta-amyloid (A*β*) and a glutamate receptor agonist [87]. Catalpol attenuates cortical primary cultured neuronal apoptosis induced by A*β*_1–42_ through a mitochondrial-dependent caspase pathway [88]. Catalpol exerts significant neuroprotection in gerbils subjected to transient global cerebral ischemia by reducing TUNEL-positive and Bax-positive cells, and increasing Bcl-2-positive cells, in the hippocampal CA1 [89, 90]. Meanwhile, catalpol protects against memory damage and energy metabolism failure by increasing the activities of SOD and glutathione peroxidase (GSH-Px), and decreasing the concentration of MDA. In addition, it elevates the activity of glutathione S-transferase (GSH–ST), glutamine synthetase (GS), and creatine kinase (CK) and decreases the activity of lactate dehydrogenase (LDH), in the brains of a mouse model of aging [91, 92]. A study has demonstrated that catalpol has satisfactory bioavailability and can penetrate the blood brain barrier (BBB) with an AUC_(CSF)_/AUC_(plasma)_ of 5.8% and half-life (t_1/2_) of 1.5 h [93].

## 5. Hypothesis

Overall, the structural abnormalities of the cortex play an important part in the pathomechanism of ADHD, which involves neuronal development disorders, neuronal apoptosis, and energy metabolism disorders. TCM theory argues that a deficiency in brain marrow induced by kidney essence results in symptoms such as hyperactivity, impulsive behavior, and attention deficit. The mechanisms of these are highly analogous to structural abnormalities in the cortex. The therapeutic principle of “supplementing the kidney and replenishing the essence” has been applied to clinical treatments of ADHD widely and proved effective and safe.

Catalpol is the active ingredient of Rehmanniae radix preparata that is neuroprotective, antiapoptotic, and can attenuate energy metabolism failure. Rehmanniae radix preparata and catalpol can be used to benefit many neuropsychiatric disorders. Considerable evidence points to the important role of Rehmanniae radix preparata and catalpol in treating ADHD; however, they have not been used in studies assessing novel treatments for ADHD.

**Figure 2:**
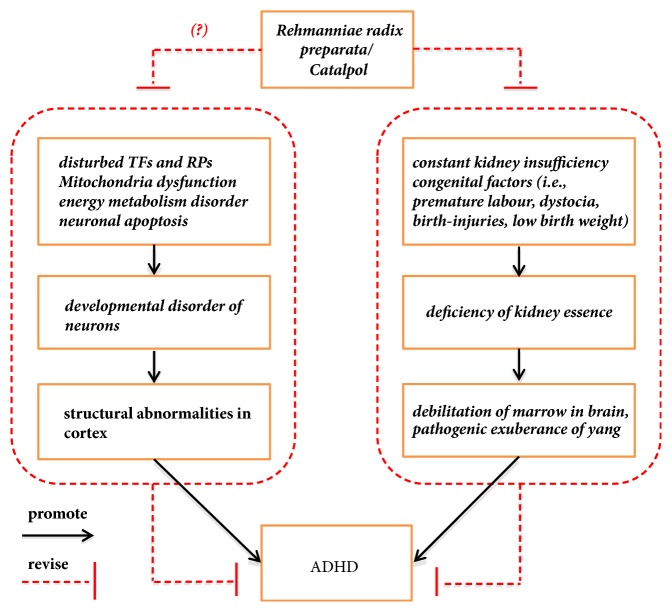
Schematic representation of our hypothesis. (a) The TCM theory argues that a physiological and pathological deficiency of the kidney essence is pivotal in the pathogenic exuberance of yang and debilitation of brain marrow, which is closely related to pathogenesis of ADHD. (b) Rehmanniae radix preparata is a classical Chinese herbal medicine that benefits the kidney essence, fills brain marrow, and enriches the blood. As the most frequently prescribed herb for treating ADHD, Rehmanniae radix preparata improves learning and memory and inhibits spontaneous activity in animal models via central inhibition, antioxidation, and antiaging. (c) Catalpol, an important active ingredient of Rehmanniae radix preparata, has several pharmacological properties, such as protecting growth factors, alleviating energy metabolism failure, and reducing neuronal apoptosis in many other neuropsychiatric disorders. (d) Dysregulation of transcription factors (TFs) and regulatory proteins (RPs), energy metabolism failure, and neuronal apoptosis are linked to neuronal developmental disorders and result in structural abnormalities in the cortex, which is associated with ADHD. Therefore, we propose that Rehmanniae radix preparata and catalpol are promising natural medicines for the treatment of ADHD.

Coupled with recent evidence, we postulate that both Rehmanniae radix preparata and catalpol are promising natural medicines for the treatment of ADHD* via* the effects of neuroprotection, antiapoptosis, and attenuating energy metabolism disorders. This may lead to the reversal of structural abnormalities in cortex (Figure 2). Based on this hypothesis, we plan to assess their effects using the SHR model. We argue that our hypothesis will lead to a diversification of research and extend the existing theories of ADHD by proposing a pathological basis for specific aspects and a new therapeutic target for the prevention and treatment of the ADHD.

## Additional Points

*Highlights*. (i) Children with ADHD show structural abnormalities in the cortex. (ii) Pathogenesis of maturational delay of cortex is in line with the TCM theory of ADHD. (iii) Rehmanniae radix preparata is the most frequently used Chinese herbal medicine to treat ADHD. (iv) Rehmanniae radix preparata may treat ADHD by alleviating structural abnormalities in the cortex.
